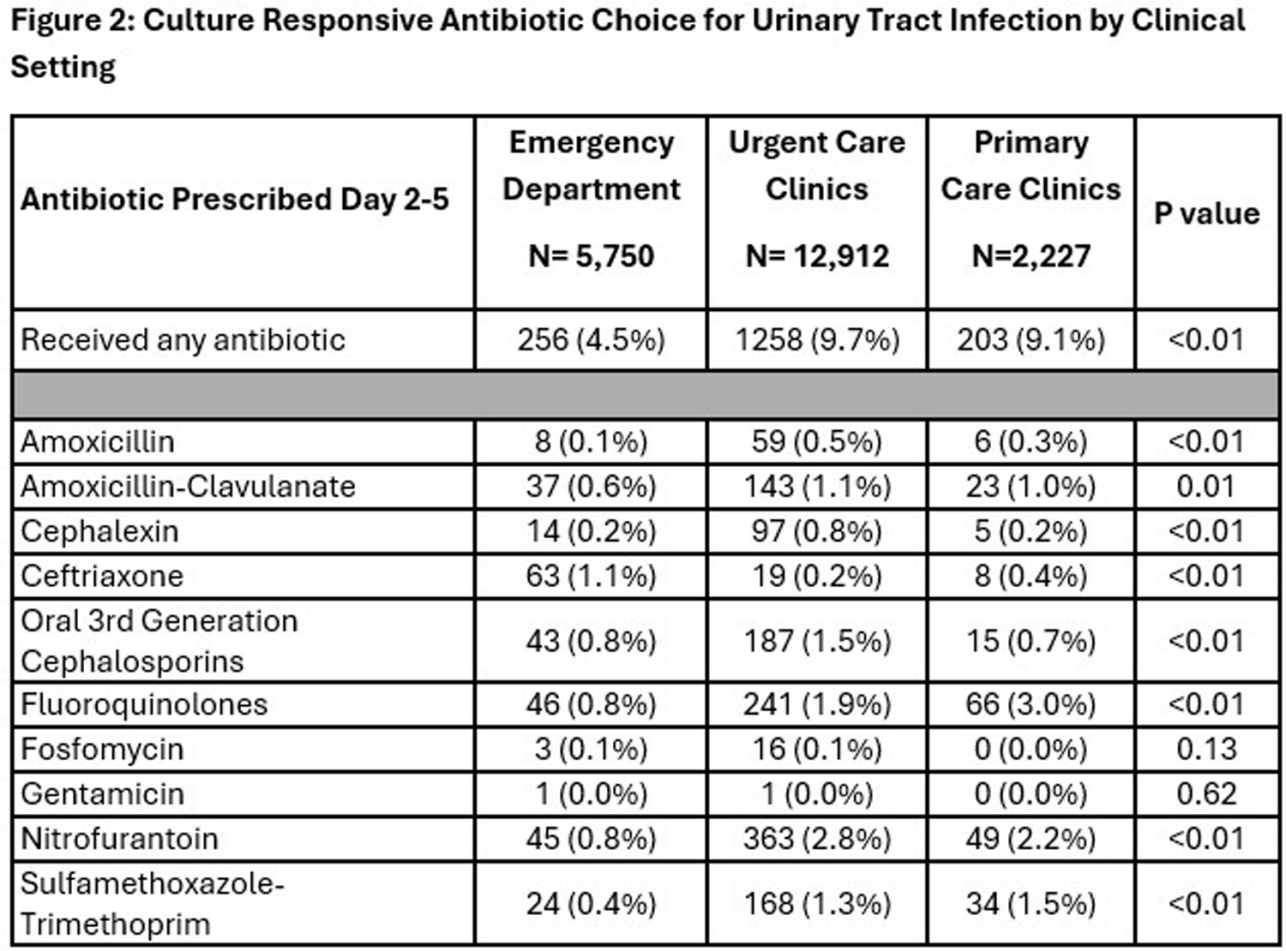# 106 Optimizing the Role of Streptococcus pneumoniae and Legionella Urinary Antigen testing

**DOI:** 10.1017/ash.2026.10523

**Published:** 2026-06-23

**Authors:** Barbara Mora, Megan Uehling, Lauren Johansen, Sharon Onguti, Rebecca Stern, Milner Staub

**Affiliations:** 1 Vanderbilt University Medical Center; 2 Vanderbilt University

## Abstract

**Background:** Urinary tract infections (UTIs) are frequently managed at outpatient settings, including primary care and urgent care (UC) clinics and emergency departments (EDs), which vary in antibiotic prescribing practices. We aimed to evaluate initial antibiotic choice and clinician responsiveness to cultures in subsequent days after the encounter by clinical setting. **Methods:** This retrospective cohort study included encounters with an order for a urinalysis with reflex urine culture from June 1, 2022 to June 30, 2024 across 29 walk-in and retail clinics (UCs), 5 EDs, and 33 primary care clinics at Vanderbilt University Medical Center. All encounters included antibiotics received with an order indication for cystitis or pyelonephritis (UTI). Encounters in which a positive urine culture was available in the preceding 6 months were excluded, as it was assumed that this data would guide antibiotic selection and therefore would not be empiric. Additionally, encounters from June to December 2022 were removed because culture data in the preceding six months could not be assessed. For each time period, antibiotic use was recorded as administered or not administered during Day 0-1 (empiric therapy) and Days 2-5 (culture-directed therapy). Patients could receive <1 antibiotic per time period per encounter. Differences in antibiotic prescribing were calculated using Chi-square and Fisher’s exact tests as indicated. **Results:** A total of 20,889 encounters were included: 5,750 (27.5%) from ED, 12,912 (61.8%) from UC, and 2,227 (10.7%) from primary care. Empiric antibiotic choice differed significantly for all antibiotics across clinical settings. Higher rates of prescribing cephalexin and third generation oral cephalosporins occurred in the ED, and fluoroquinolones in primary care, while lower use of nitrofurantoin was noted in the ED (Figure 1). A total of 256/5,750 (4.5%) patients at the ED, 1,258/12,912 (9.7%) at UCs, and 203/2,227 (9.1%) at primary care received at least one subsequent antibiotic (Figure 2). Compared to the ED, a higher percentage of both UC and primary care patients received culture-responsive antibiotics. Antibiotic choice patterns were similar for empiric and culture-responsive antibiotics. **Conclusion:** Higher percentages of culture-responsive antibiotic prescriptions were noted for primary care and UC compared to ED. At the time of this study, UCs had dedicated nurses following up results, a system more closely mirroring procedures at primary care clinics, likely resulting in more follow up antibiotic changes. Significant differences were seen across all settings for both empiric and culture-responsive antibiotic choices and represent future targets for antimicrobial stewardship.

**Figure:**
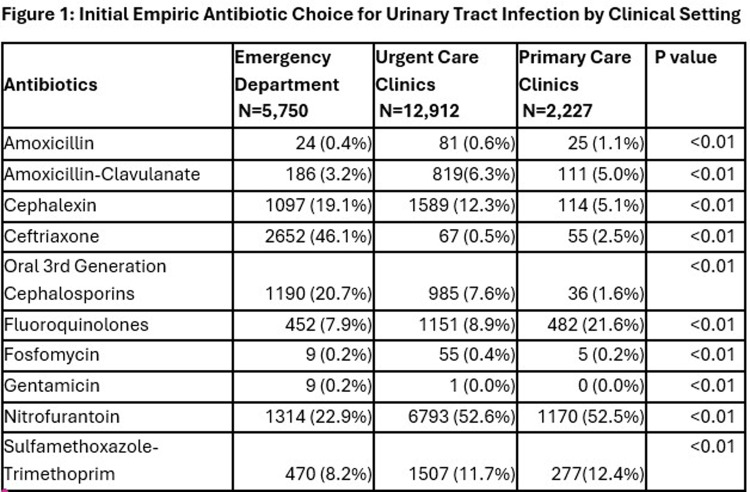

**Figure:**